# Acupuncture Versus Oral Medications for Acute/Subacute Non-Specific Low Back Pain: A Systematic Review and Meta-Analysis

**DOI:** 10.1007/s11916-023-01201-7

**Published:** 2024-01-08

**Authors:** Huize Lin, Xiang Wang, Yixuan Feng, Xiaoxu Liu, Lanping Liu, Kexin Zhu, Jianghong Shen, Pingping Zhang, Jinna Yu, Tao Yang

**Affiliations:** 1 Department of Acupuncture-Moxibustion, Guang’anmen Hospital, China Academy of Chinese Medical Sciences, Beijing, 100053 China; 2 Department of Acupuncture-Moxibustion, Institute of Acupuncture and Moxibustion, China Academy of Chinese Medical Sciences, Beijing, 100053 China

**Keywords:** Non-specific low back pain, Acupuncture, Acute, Subacute, Systematic review, Meta-analysis

## Abstract

**Purpose of Review:**

Pharmacologic intervention do not always achieve benefits in the treatment of acute/subacute non-specific low back pain (NSLBP). We assessed efficacy and safety of acupuncture for acute/subacute NSLBP as alternative treatment.

**Recent Findings:**

We searched PubMed, Web of Science, Embase, Cochrane Library, Scopus, Epistemonikos, CNKI, Wan Fang Database, VIP database, CBMLD, CSTJ, clinical trials, EUCTR, World WHO ICTRP, and ChiCTR for randomized controlled trials, cross-over studies, and cohort studies of NSLBP treated by acupuncture versus oral medication from inception to 23th April 2022. A total of 6 784 records were identified, and 14 studies were included 1 263 participants in this review. The results of the meta-analysis indicated that acupuncture therapy was slightly more effective than oral medication in improving pain (*P* < 0.00001, *I*^2^ = 92%, MD = −1.17, 95% CI [−1.61, −0.72]). According to the results of the meta-analysis, acupuncture therapy exhibited a significant advantage over oral medication with a substantial effect (*P* < 0.00001, *I*^2^ = 90%, SMD = −1.42, 95% CI [−2.22, −0.62]). Based on the results of the meta-analysis, acupuncture therapy was associated with a 12% improvement rate compared to oral medication in patients with acute/subacute NSLBP (*P* < 0.0001, *I*^2^ = 54%, RR = 1.11, 95% CI [1.05, 1.18]).

**Summary:**

Acupuncture is more effective and safer than oral medication in treating acute/subacute NSLBP. This systematic review is poised to offer valuable guidance to clinicians treating acute/subacute NSLBP and potentially benefit the afflicted patients.

**Registration:**

This review was registered in PROSPERO (http://www.crd.york.ac.uk/prospero) with registration number CRD42021278346.

**Supplementary Information:**

The online version contains supplementary material available at 10.1007/s11916-023-01201-7.

## Introduction

Non-specific low back pain (NSLBP) lacks a precisely defined pathoanatomical etiology, constituting the predominant variety, responsible for 90–95% of all cases of lower back pain [1]. Furthermore, low back pain (LBP) exhibits a worldwide point prevalence of 9.4% [2], and a 1-month prevalence of 30.8% [3]. Notably, 60% of cases of acute NSLBP progress to a chronic state [4]. LBP represents the foremost chronic health issue, compelling older employees to retire prematurely and causing more work absences than the combination of heart disease, diabetes, hypertension, neoplasms, respiratory diseases, and asthma [5]. NSLBP affects individuals across all age groups, with a notable impact on the elderly [6••], and has consistently imposed the most substantial burden on global healthcare economics, both directly and indirectly, for the past three decades [2]. For instance, in the USA, the estimated economic burden due to reduced productivity in individuals with LBP reached $23.5 billion [7]. Hence, addressing the treatment and prediction of acute/subacute NSLBP assumes paramount importance.

Guidelines advocate reassuring patients regarding a favorable prognosis and offering advice on the limited use of nonsteroidal anti-inflammatory drugs (NSAIDs) and weak opioids for brief durations to manage non-specific, short-term lower back pain [6••]. Concurrently, it is noteworthy that certain drugs may be associated with a heightened occurrence of adverse events; therefore, clinicians and patients are advised to exercise caution in their approach to the utilization of analgesic medications [8•].

Acupuncture is prominently featured as a therapeutic modality in the guidelines for managing LBP [9]. While multiple prior meta-analyses [10] have demonstrated the potential efficacy of acupuncture in alleviating clinical symptoms of NSLBP, there has been a notable absence of studies that have specifically scrutinized the effectiveness and safety of acupuncture compared to pharmacological interventions for acute/subacute NSLBP.

Clinicians accorded the second-highest priority to the establishment of clinical practice guidelines for acupuncture-moxibustion in the management of NSLBP [11••]. To comprehensively assess the clinical efficacy and safety of acupuncture in the context of acute/subacute NSLBP, a novel meta-analysis was undertaken, addressing limitations identified in prior systematic reviews, including limited literature coverage, lenient inclusion criteria, and outdated research methodologies. In this systematic review, we have synthesized the evidence regarding the efficacy and safety of acupuncture in comparison to oral medications for patients afflicted with acute and/or subacute non-specific LBP.

## Methods

The review protocol was registered in PROSPERO (http://www.crd.york.ac.uk/prospero) with registration number CRD42021278346. Furthermore, the systematic review was conducted in accordance with performed following the preferred reporting items for systematic reviews and meta-analysis protocols (PRISMA) guidelines [12].

### Criteria for Considering Reviews for Inclusion

We included all randomized controlled trials (RCTs), cross-over studies, and cohort studies on acupuncture interventions for individuals with acute or subacute NSLBP without language limitation. To maintain the rigor of this systematic review, quasi-RCTs, reviews, case reports, experimental studies, expert experience, letters, animal experiments, studies with incomplete data, studies without comparable baselines, and duplicate publications were all excluded.

### Types of Participants

LBP is defined as a primary area of pain primarily located between the 12 rib and gluteal fold, with or without associated leg pain [13]. In this systematic review, the study population consisted of individuals experiencing acute (0–4 weeks) or subacute (4–12 weeks) NSLBP. Excluded were cases involving spinal stenosis (back and leg pain associated with narrowing of the spinal canal), LBP caused by known structural or pathological processes (e.g., nerve root compression, osteoporosis, fractures, infection, neoplasm, metastasis) or specific medical conditions (e.g., pregnancy, inflammatory disease) [13]. There were no restrictions based on age, sex, and geographic region.

### Types of Interventions and Comparisons

Trials that meet the criteria comparing acupuncture with the oral medication were eligible. In this review, acupuncture specifically encompassed acupuncture therapy (manual/hand acupuncture and electro-acupuncture), necessitating the insertion of acupuncture needle into acupoints or pain points [14]. Oral medications included NSAIDs and paracetamol (acetaminophen), muscle relaxants, benzodiazepines, opioid analgesics, antidepressants, anticonvulsants, and systemic corticosteroids. No restrictions were imposed on the route of administration or dose. Additionally, acupuncture in combination with any form of pharmacologic intervention and another acupuncture treatment method as control groups was excluded from the control group.

### Types of Outcome Measures

The primary outcome was pain intensity, assessed using the visual analog scale (VAS), numerical rating scale (NRS), or other validated scales. The secondary outcome were functional status, improvement rate, and safety.

Functional status was assessed through the Oswestry disability index (ODI), Roland-morris disability questionnaire (RMDQ), Lumbar range of motion (LROM), and Schober test. The improvement rate could be defined according to specific criteria. All adverse and acupuncture-related adverse events were analyzed for safety.

The analysis time point was set at the end of the conclusion of all scheduled treatment sessions.

### Information Sources and Search

We searched PubMed, Web of Science, Embase, Cochrane Library, Scopus, Epistemonikos, China National Knowledge Infrastructure (CNKI), Wan Fang database, VIP (China Academic Journals) database, Chinese Biomedical Literature Database (CBMLD), China Science and Technology Journal Database (CSTJ), Clinical Trials, European Union Clinical Trial Register (EUCTR), World Health Organisation International Clinical Trials Registry Platform (WHO ICTRP), and Chinese Clinical Trial Registry (ChiCTR) from the initial issue to 23th April 2022 using Chinese and English language and publication status.

This search strategy was modified as required for other electronic databases. The research strategy used in PubMed is in Table 1.

**Table 1:** The search strategy used in PubMed

Number	Search strategy for Pubmed
#1	“Low back pain” [mesh]
#2	“Lower back pain” OR “low back ache” OR “low backache” OR “postural low back pain” OR “recurrent low back pain” OR “mechanical low back pain” OR “lumbago” OR “lumbodynia” OR “lumbar pain” OR “osphyalgia” OR “lumbar myalgia” OR “lumbar sprain” OR “lumbosacral sprain” OR “lumbosacral strain” OR “sacroiliac sprain” OR “sacroiliac strain” OR “muscular strain of the lumbar region” OR “third lumbar transverse process syndrome” OR “lumbar muscle fasciitis” OR “lumbar gluteal myofascitis” OR “NLBP” OR “ANLBP” OR “NSLBP”
#3	“acupuncture” OR “electro-acupuncture” OR “electro acupuncture” OR “body acupuncture” OR “body needling” OR “body needle” OR “hand acupuncture” OR “manual needling” OR “hand needle” OR “manual acupuncture” OR “acupuncture point” OR “acupoint”
#4	“randomised controlled trial” OR “controlled clinical trial” OR “randomised” OR “randomly” OR “trial” OR “group” OR “cross-over study” OR “cohort study”
#5	#1 OR #2
#6	#5 AND # 3 AND #4

### Study Selection, Data Extraction and Management, and Methodological Quality Assessment

Independently, we screened the titles and abstracts of all identified records, adhering to the criteria for study inclusion. Moreover, two reviewers (HZ-L and XW) autonomously obtained the full texts of studies meeting the eligibility criteria and subsequently conducted a reevaluation to affirm their inclusion. The retrieved literature was managed using EndNote 20 software, and any duplicate citations were meticulously eliminated.

In the case of included studies, essential study details, population characteristics, intervention specifics, outcomes of interest, and results were independently extracted and subsequently cross-verified by two reviewers (HZ-L and XW) using an Excel-based form. Additionally, two reviewers (HZ-L and XW) autonomously evaluated potential biases, including selection bias, performance bias, detection (or measurement) bias, attrition bias, reporting bias, and other registered or unregistered biases, in each included trial by employing a rating system encompassing categories of “yes,” “no,” and “unclear.”

In cases where data appeared ambiguous, we communicated with authors via email or telephone to obtain the necessary information for eligibility confirmation. In instances of disagreements during study selection, data extraction, data management, or methodological quality assessment, resolution was achieved through discussion between the two reviewers (HZ-L and XW), or, if required, by seeking the input of a third independent reviewer (JN-Y).

### Data Synthesis and Analysis

Meta-analysis was conducted using RevMan V.5.3.5. When the statistical heterogeneity was low (*P* ≥ 0.1, or *I*^2^ ≤ 50%), we used the fixed-effect model to combine the data. In contrast, we used the random-effect model when the statistical heterogeneity was high (*P* < 0.1, or *I*^2^ > 50%). In addition, for continuous data, the weighted mean difference (WMD) was selected when the effect of the same intervention was measured using the same method or unit, together with a 95% confidence interval (CI). The standardized mean difference (SMD) was applied when the outcome of studies was measured in different ways. Finally, the risk ratio was selected for dichotomous data, together with the 95% CI. Statistical significance was set at *P* ≤ 0.05.

We classified the size of the effect for mean between-group difference for the outcome pain and function based on the minimum clinically important difference (MCID) [15] (Table 2).

**Table 2:** The minimum clinically important difference

	**Trivial effect**	**Small effect**	**Moderate effect**	**Large effect**
VAS	< 0.5 cm	0.5~1 cm	1~2 cm	> 2 cm
NRS	< 0.5 points	0.5~1 points	1~2 points	> 2 points
ODI	< 5 points	5~10 points	10~20 points	> 20 points
RMDQ	< 1 point	1~2 points	2~5 points	> 5 points
RR (Improvement rate)	0.7~0.8 or 1.2~1.4	0.4~0.6 or 1.5~2.9	0.1~0.3 or 3.0~9.9	< 0.1 or > 10
SMD	< 0.2	0.2~0.5	0.5~0.8	> 0.8

The certainty of evidence on effect estimates used the grading of recommendation assessment, development, and evaluation (GRADE) approach. The evidence grading method for each outcome included the risk of bias, inconsistency of results, indirectness of evidence, imprecision, and publication bias. In addition, the confidence of the evidence was designated as high, moderate, low, or very low.

## Results

### Study Selection and Characteristics

**Fig. 1:**
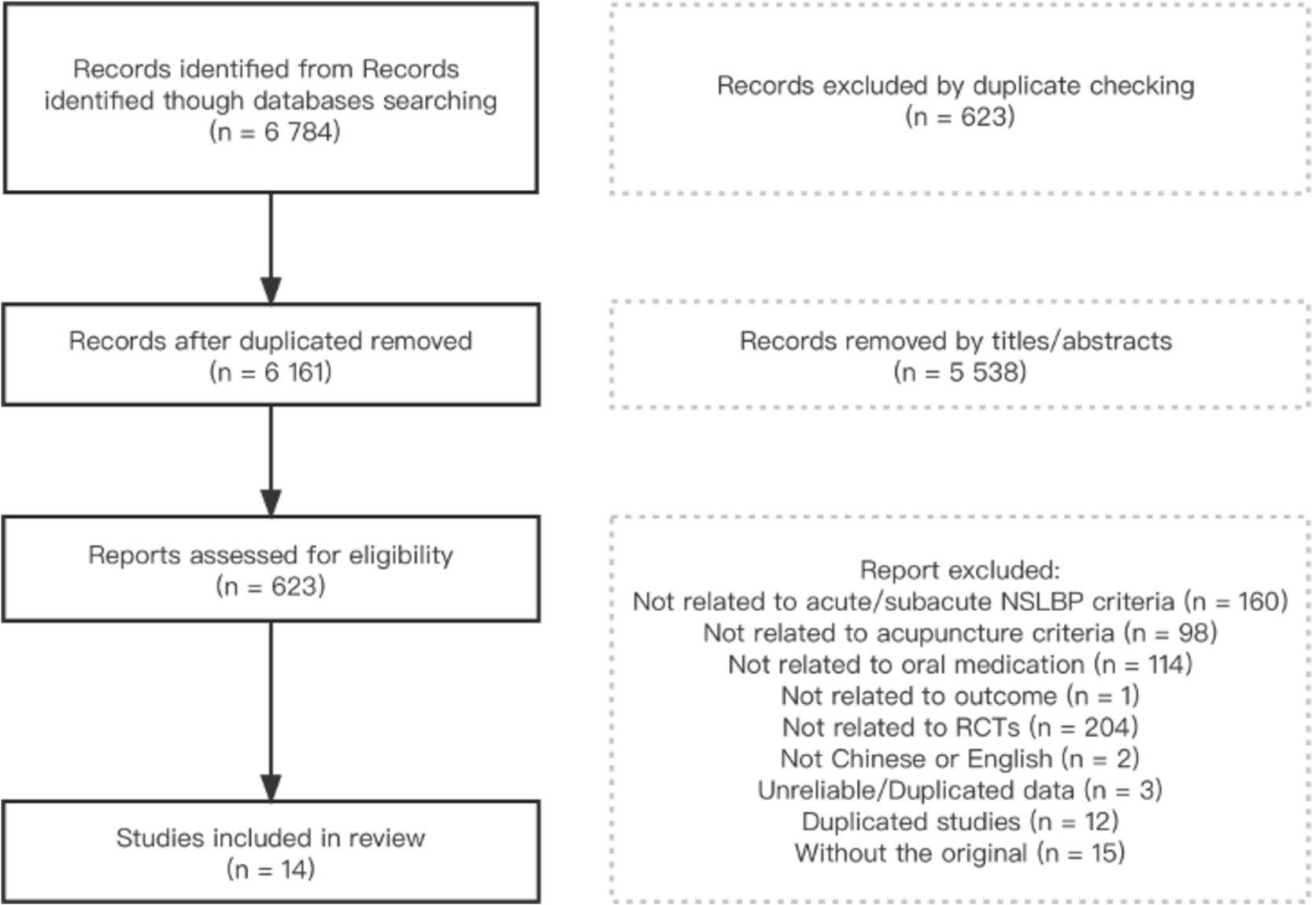
Article selection process

A total of 6 784 records were identified, and 14 studies [16–29] were included with 1263 participants in this review (Fig. 1). We contacted the authors of one study [16] via email to clarify information not adequately reported. After responding, we used the data as reported in this review. All 14 studies mentioned that there was no significant difference in general data between the observation group and the control group (*P* > 0.05), which was comparable.

The intervention was classified to the different treatment method and acupuncture prescription. Based on the treatment classification, among the included 14 studies, nine studies [16, 20–24, 26, 28] selected distal acupoints combined with lumbar movement (referred to as motion-style acupuncture treatment). We categorized the included studies into four types and performed the subgroup analyses based on stimulating acupuncture points while simultaneously instructing patients to engage in specific movements (Table 3). Only one study used LROM as measurement [27] and two studies used the Schober test as measurement [19, 25]; we combined them into no movement group for analysis. Among the studies, common oral medication for acute/subacute NSLBP include NSAIDs (such as diclofenac sodium [17, 21, 26], ibuprofen [22, 23], loxoprofen [16], meloxicam [18, 20, 24, 25, 27, 28], nimesulide [29]) as well as muscle relaxants (eperisone) [19]. With studies, common distal acupuncture points included EX-UE 7 [16, 20, 23, 24, 28], GV 26 plus SI 3 [28, 29], SI 6 [26], and distal points of the meridian (SI 6, BL 2, GV 26) [21]. The remaining five studies selected different proximal points [19], distal points [17, 18], or proximal combined with distal points [22, 29] (Table 4).

**Table 3:** Subgroup and Intervention method

Subgroup		Intervention method
Movement group	Multiple acupuncture session	Acupoints, multiple treatment session, combined with movement.
	Acupuncture only once	Acupoints, only once treatment, combined with movement.
No movement group	Electroacupuncture on Ashi points only	Electroacupuncture on Ashi points only, multiple treatment session, no movement.
	Acupoint selection of the physician’s personal clinical experience	Acupuncture combined with acupoints, multiple treatment session, no movement.

**Table 4:** Characteristics of Included Studies

**Studies**	**Age (mean), y**		**Course, d**		**Intervention group (sample size)**	**Control group (sample size)**	**Frequency of acupuncture**	**Treatment duration, d**	**Outcomes of interest**	**Time point included in the analysis**	**Follow-up, d**
	**Intervention group**	**Control group**	**Intervention group**	**Control group**							
Liu et al. [16]	43	43	/	/	Movement + Yaotong (EX-UE 7) and Ashi point (26)	NSAIDs: loxoprofen (20)	3times/week	7	VAS, Improvement rate	7d	/
Fan and Wu [17]	44	43	1.4	1.5	No movement (EA) + Houxi (SI 3) to Hegu (LI 4), Jiaji (EX-B 2) and Ashi points (60)	NSAIDs: diclofenac sodium (60)	1/d	7	Improvement rate	7d	7d
Wu et al. [18]	45	46	2~7	2~7	No movement (EA) + Houxi (SI 3) (150)	NSAIDs: meloxicam (150)	1/d	14	Improvement rate	14d	30d
Qu et al. [19]	46	46	1	6~7	No movement (EA) + Shenshu (BL 23), Dachangshu (BL 25), Weizhong (BL 40), and Ashi points (20)	Muscle relaxants: eperisone (20)	1/d	1	VAS, Schober test, Improvement rate	1d	/
Li and Liu [20]	/	/	/	/	Movement + Yaotong (EX-UE 7) (35)	NSAIDs: meloxicam (31)	1/d	1	Improvement rate	1d	2d
Shang et al. [21]	42	43	1~5	1~5	Movement + Yanglao (SI 6), Cuanzhu (BL 2), and Shuigou (GV 26) (30)	NSAIDs: diclofenac sodium (30)	1/d	3	VAS, ODI, Improvement rate	3d	/
		43	1~5	1~5	No movement + Ashi points (30)	NSAIDs: diclofenac sodium (30)	1/d	3	VAS, ODI, Improvement rate	3d	/
Gao et al. [22]	/	/	3	3	Movement + Lumbar Pain Point (BL 23), and Ashi points (36)	NSAIDs: ibuprofen (36)	3/d	3	Improvement rate	3d	/
Du [23]	38	37	/	/	Movement + Yaotong (EX-UE 7) (30)	NSAIDs: ibuprofen (30)	1/d	3	VAS, LROM, Improvement rate	120 min	/
Jin and Chen [29]	45	45	2~7	2~7	Movement + Shuigou (GV 26), Houxi (SI 3), Shenshu (BL 23), Dachangshu (BL 25), and Weizhong (BL 40) (40)	NSAIDs: nimesulide (40)	1/d	2	Improvement rate	2d	/
Wang et al. [25]	/	/	1~2		No movement (EA) + Ashi point (40)	NSAIDs: meloxicam (40)	1/d	6	VAS, Schober test, Improvement rate	6d	/
Xu [26]	/	/	/	/	Movement + Yanglao (SI 6) (40)	NSAIDs: diclofenac sodium (50)	1/d	5	RMDQ, Improvement rate	5d	10d
Huang [24]	44	46	2	2	Movement + Yaotong(EX-UE 7) (50)	NSAIDs: meloxicam (30)	1/d	5	VAS, LROM, Improvement rate, RMDQ	5d	/
Zhao et al. [28]	/	/	/	/	Movement + Shuigou(GV 26), Yaotong(EX-UE 7), and Houxi (SI 3) (30)	NSAIDs: meloxicam (22)	1/d	3	Improvement rate	3d	/
Sun [27]	51	51	2~7	2~7	No movement + EA Chengshan (BL 57) (23)	NSAIDs: meloxicam (32)	1/d	5	VAS, LROM, Improvement rate, RMDQ	5d	/
	50	51	2~7	2~7	No movement + EA Ashi points (32)	NSAIDs: meloxicam (32)	1/d	5	VAS, LROM, Improvement rate, RMDQ	5d	/

### Quality of the Included Studies

**Fig. 2:**
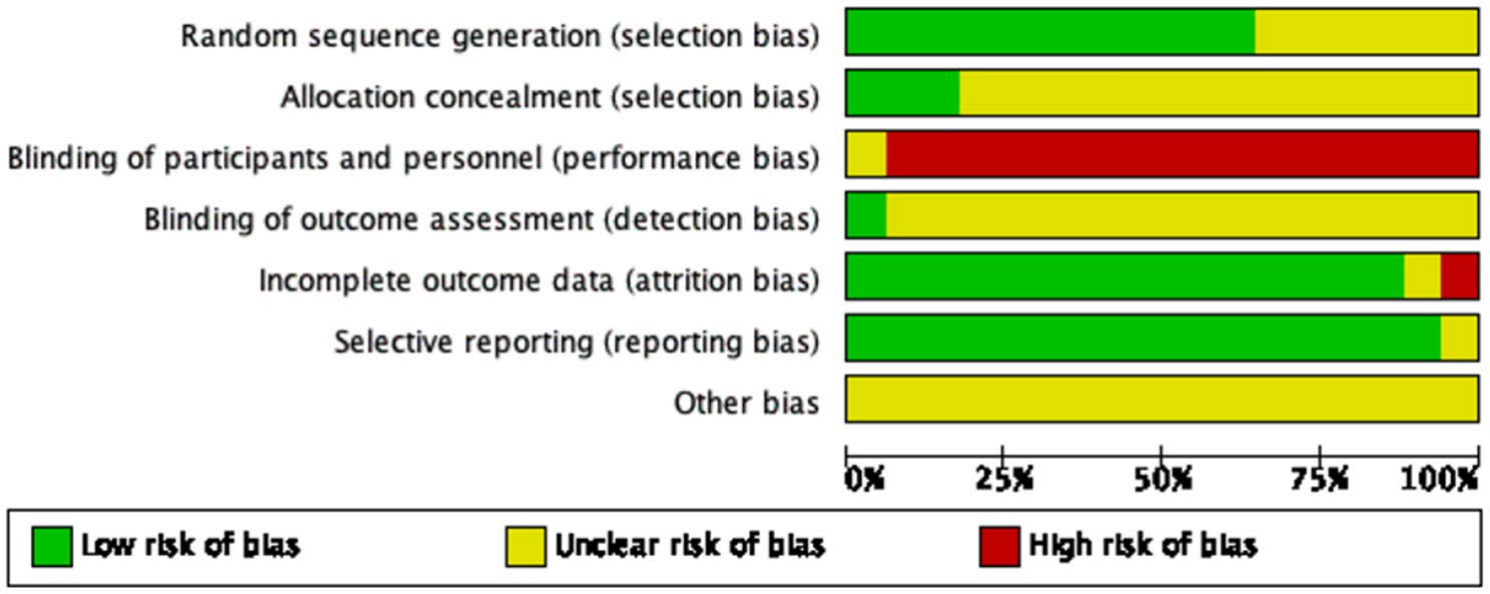
Quality of the included studies

Adequate methods for generating random sequences were reported in ten articles [16–19, 21–26] utilizing the random number table technique. In four studies [20, 27–29], the process of random sequence generation was not mentioned, leading us to evaluate them with an unclear risk of bias. Two studies provided detailed descriptions of allocation concealment methods [23, 26], whereas the remaining studies did not specify the use of allocation concealment, resulting in an assessment of unclear risk of bias. Blinding of acupuncture therapists was not feasible in any of the studies, leading to a high-risk bias rating. One study detailed the blinding of outcome evaluation and assessment procedures [17] resulting in a low-risk bias rating. Three studies [17, 18, 23] were deemed to have a high risk of attrition bias due to a significant number of subjects who dropped out of the studies. In the case of all remaining studies, the protocol was unavailable, resulting in an assessment of an unclear risk of reporting bias (Fig. 2).

### Effects Estimates

#### Pain

The results of the meta-analysis indicated that acupuncture therapy was slightly more effective than oral medication in improving pain (*P* < 0.00001, *I*^2^ = 92%, MD = −1.17, 95% CI [−1.61, −0.72]; 9 studies, 468 participants; moderate effect, very low-quality evidence).

Subgroup analysis results revealed that the movement group (involving multiple acupuncture sessions) (with acupuncture administered 3–6 times during lumbar movement) (*P* < 0.00001, *I*^2^ = 47%, MD = −2.42, 95% CI [−3.03, −1.80]; 3 studies, 151 participants; large effect, very low-quality evidence) exhibited better outcomes than the no movement group (involving only electroacupuncture at Ashi points) (*P* < 0.00001, *I*^2^ = 0%, MD = −0.42, 95% CI [−0.57, −0.27]; 2 studies, 127 participants; trivial effect, low-quality evidence) and the no movement group (acupoint selection of the physician’s personal clinical experience) (*P* = 0.61, *I*^2=^0%, MD = −1.10, 95% CI [−1.24, −0.97]; 3 studies, 133 participants; moderate effect, very low-quality evidence).

No significant difference was observed between the effects of the movement group (involving acupuncture administered only once) and the oral medication group (*P* = 0.36, *Z* = 0.92, MD = −0.32, 95% CI [−1.00, −0.36]; 1 study, 57 participants; trivial effect, low-quality evidence) (Table 5; Fig. 1 in the Supplementary Material).

**Table 5:** Result of meta-analysis and evidence grade

**Outcome**	**Result**	**No. of studies (participants)**	**Grade of evidence**	**Favor**
**Acupuncture group vs oral medication**				
Pain	*P* < 0.00001, *I*^2^ = 92%, MD = −1.17, 95% CI (−1.61, −0.72]	9 studies, 468 participants	Moderate effect, very low-quality evidence	Acupuncture group
Function (RMDQ/ODI)	*P* = < 0.00001, *I*^2^ = 90%, SMD = −1.42, 95% CI (−2.22, −0.62]	6 studies, 346 participants	Large effect, very low-quality evidence	Acupuncture group
Improvement rate	*P* < 0.0001, *I*^2^ = 54%, RR = 1.11, 95% CI (1.05, 1.18]	14 studies, 1078/1028 participants	Trivial effect, very low-quality evidence	Acupuncture group
**Movement group (multiple acupuncture session) vs oral medication**				
Pain	*P* < 0.00001, *I*^2^ = 47%, MD = −2.42, 95% CI (−3.03, −1.80]	3 studies, 151 participants	Large effect, very low-quality evidence	Movement group (multiple acupuncture session)
Function (RMDQ/ODI)	*P* < 0.00001,* I*^2^ = 90%, SMD = −1.37, 95% CI (−2.39, 0.35]	3 studies, 205 participants	Large effect, very low-quality evidence	Movement group (multiple acupuncture session)
Improvement rate	*P* = 0.26, *I*^2^ = 22%, RR = 1.19, 95% CI (1.09, 1.30]	7 studies, 385/448 participants	Trivial effect, low- quality evidence	Movement group (multiple acupuncture session)
**Movement group (acupuncture only once) vs oral medication**				
Pain	*P* = 0.36, Z = 0.92, MD = − 0.32, 95% CI (−1.00, −0.36]	1 study, 57 participants	Trivial effect, low- quality evidence	Movement group (acupuncture only once)
Improvement rate	*P* = 0.93, *I*^2^ = 0%, RR = 1.09, 95% CI (0.94, 1.27]	2 studies, 76/85 participants	Small effect, low- quality evidence	Movement group (acupuncture only once)
**No movement group (electroacupuncture on Ashi points only) vs oral medication**				
Pain	*P* < 0.00001, *I*^2^ = 0%, MD = −0.42, 95% CI (−0.57, −0.27]	2 studies, 127 participants	Trivial effect, low- quality evidence	No movement group (electroacupuncture on Ashi points only)
Function (RMDQ/ODI)	*P* < 0.00001, SMD = 0.00, 95% CI (−0.60, 0.60]	1 study, 48 participants	Trivial effect, very low-quality evidence	No movement group (electroacupuncture on Ashi points only)
Improvement rate	*P* = 0.43, *I*^2^ = 0%, RR = 1.02, 95% CI (0.96, 1.10]	2 studies, 123/127 participants	Trivial effect, low- quality evidence	No movement group (electroacupuncture on Ashi points only)
**No movement group (acupoint selection of the physician’s personal clinical experience) vs oral medication**				
Pain	*P* = 0.61, *I*^2^ = 0%, MD = −1.10, 95% CI (−1.24, −0.97]	3 studies, 133 participants	Moderate effect, very low-quality evidence	No movement group (acupoint selection of the physician’s personal clinical experience)
Function (RMDQ/ODI)	*P* = 0.21, *I*^2^ = 37%, SMD = −2.23, 95% CI (−2.92, 1.54]	2 studies, 93 participants	Large effect, very low-quality evidence	No movement group (acupoint selection of the physician’s personal clinical experience)
Improvement rate	*P* = 0.03, *I*^2^ = 63%, RR = 1.09, 95% CI (0.99, 1.21]	5 studies, 494/548 participants	Trivial effect, very low-quality evidence	No movement group (acupoint selection of the physician’s personal clinical experience)
**No movement group vs oral medication**				
Function (LROM)	*P* < 0.00001, *I*^2^ = 100%, MD = 33.92, 95% CI (−19.03, 86.86]	1 study, 95 participants	Trivial effect, very low-quality evidence	No statistically different
Function (Schober test)	*P* = 0.009, *I*^2^ = 85%, MD = 1.27, 95% CI (−0.77,3.31]	2 studies, 120 participants	Trivial effect, very low-quality evidence	No statistically different

#### Function

1. RMDQ
  According to the results of the meta-analysis, acupuncture therapy exhibited a significant advantage over oral medication with a substantial effect (*P* < 0.00001, *I*^2^ = 90%, SMD =  − 1.42, 95% CI [− 2.22, − 0.62]; 6 studies, 346 participants; large effect, very low-quality evidence).
  Subgroup analysis results indicated that the movement group (involving multiple acupuncture sessions) (*P* < 0.00001, *I*^2^ = 90%, SMD =  − 1.37, 95% CI [− 2.39, 0.35]; 3 studies, 205 participants; large effect, very low-quality evidence) and the no movement group (acupoint selection of the physician’s personal clinical experience) (*P* = 0.21, *I*^2^ = 37%, SMD =  − 2.23, 95% CI [− 2.92, 1.54]; 2 studies, 93 participants; large effect, very low-quality evidence) were both superior to the oral medication group. However, no significant difference was observed between the no movement group (involving electroacupuncture at Ashi points only) (*P* < 0.00001, SMD = 0.00, 95% CI [− 0.60, 0.60]; 1 study, 48 participants; trivial effect, very low- quality evidence) and the oral medication group (Table 5; Fig. 2 in the Supplementary Material).
2. In terms of improving LROM in patients with acute/subacute NSLBP, there was no significant difference between acupuncture therapy and oral medication (*P* < 0.00001, *I*^2^ = 100%, MD = 33.92, 95% CI [−19.03, 86.86]; 1 study, 95 participants; trivial effect, very low-quality evidence) (Table 5; Fig. 3 in the Supplementary Material).
3. In terms of improving Schober test scores in patients with acute/subacute NSLBP, there was no significant difference between acupuncture therapy and oral medication (*P* = 0.009, *I*^2^ = 85%, MD = 1.27, 95% CI [−0.77,3.31]; 2 studies, 120 participants; trivial effect, very low-quality evidence) (Table 5; Fig. 4 in the Supplementary Material).

#### Improvement Rate

Based on the results of the meta-analysis, acupuncture therapy was associated with a 12% improvement rate compared to oral medication in patients with acute/subacute NSLBP (*P* < 0.0001, *I*^2^ = 54%, RR = 1.11, 95% CI [1.05, 1.18]; 14 studies, 1078/1028 participants; trivial effect, very low-quality evidence).

Subgroup analysis results revealed that the movement group (involving multiple acupuncture sessions) (*P* = 0.26, *I*^2^ = 22%, RR = 1.19, 95% CI [1.09, 1.30]; 7 studies, 385/448 participants; trivial effect, low-quality evidence) and the no movement group (acupoint selection of the physician’s personal clinical experience) (*P* = 0.03, *I*^2^ = 63%, RR = 1.09, 95% CI [0.99, 1.21]; 5 studies, 494/548 participants; trivial effect, very low-quality evidence) exhibited better outcomes than the oral medication group. However, there was no significant difference between the movement group (involving acupuncture administered only once) (*P* = 0.93, *I*^2^ = 0%, RR = 1.09, 95% CI [0.94, 1.27]; 2 studies, 76/85 participants; small effect, low-quality evidence), the no movement group (involving electroacupuncture at Ashi points only) (*P* = 0.43, *I*^2^ = 0%, RR = 1.02, 95% CI [0.96, 1.10]; 2 studies, 123/127 participants; trivial effect, low-quality evidence), and the oral medication group (Table 5; Fig. 5 in the Supplementary Material).

#### Safety

With studies, among which ten did not mention adverse events or reactions while four studies provided descriptions of adverse reactions:

1. Fan and Wu [17]: The acupuncture group and the oral medication group each comprised 60 cases. No adverse reactions were reported in either group.
2. Xu [26]: The acupuncture group and the oral medication group each included 50 cases. In the oral medication group, 2 cases experienced upper abdominal pain and discomfort as adverse reactions; the acupuncture group did not report any adverse reactions.
3. Sun et al. [27]: There were a total of three groups: the BL 57 group with 32 cases, the Ashi point group with 31 cases, and the medication group with 32 cases. In the Ashi point group, one case experienced mild fainting during acupuncture. Following needle removal, the BL 57 group and the Ashi point group reported 3 and 5 cases of minor bleeding, respectively. The oral medication group did not provide reports of adverse reactions.
4. Huang [24]: Both the acupuncture group and the oral medication group had 30 cases. In the acupuncture group, 2 cases experienced fainting, and 1 case had a subcutaneous hematoma. The oral medication group did not report any adverse reactions.

### Sensitivity Analysis

After sequentially excluding each trial from the meta-analysis, there were no substantial differences between the pre-sensitivity and postsensitivity pooled effects for the effective rate, motor dysfunction, and the lumbar range of motion-Schober.

## Discussion

This study conducted a meta-analysis involving 14 RCTs to assess the specific effects of acupuncture (filiform needle therapy) in comparison to oral medication for the management of acute/subacute NSLBP. The results revealed that acupuncture demonstrated a slight effect on pain reduction, improvement rate, and motor function among patients with acute/subacute NSLBP when compared to oral medication. Notably, acupuncture therapy combined with lumbar movement (comprising three to six sessions) exhibited a superior effect on pain intensity improvement (moderate effect, very low-quality evidence) compared to other acupuncture method. Regarding the improvement rate, acupuncture therapy demonstrated a 12% improvement over oral medication. Regarding motor dysfunction, acupuncture therapy outperformed oral medication (trivial effect, very low-quality evidence). As for lumbar range of motion and Schober test scores, no significant difference was observed between acupuncture therapy and oral medication (large effect, very low-quality evidence). It is noteworthy that only three RCTs reported treatment-related adverse reactions, preliminarily suggesting that acupuncture is relatively safe in the treatment of acute/subacute NSLBP patients. Furthermore, all included RCTs solely reported short-term efficacy, as the long-term effects remain undetermined.

For a precise evaluation of acupuncture’s effectiveness, we concentrated on commonly used acupuncture therapy (acupuncture and electroacupuncture treatments) compared to oral medication. Common oral medications for acute/subacute NSLBP encompass NSAIDs, muscle relaxants, and opioids. Nevertheless, it is essential to note that no opioid-controlled studies were identified in our literature search. Meanwhile, for the sake of eliminating potential confounding variables and better distinguishing acupuncture’s therapeutic effects from adjunctive treatments, we excluded studies that utilized sham acupuncture needling as comparison [30] and acupuncture combined with other treatment methods as an intervention. This, in part, contributed to a more accurate assessment of acupuncture’s efficacy in clinical settings.

By analyzing the included studies, we attempted to ascertain whether incorporating physical activity (lumbar movement) into acupuncture treatments could yield more effective results. Subgroup analyses were conducted based on whether acupuncture was combined with lumbar movement. Motion-style acupuncture treatment involves stimulating acupuncture points while simultaneously instructing patients to engage in specific movements. This approach primarily benefits patients with conditions characterized by movement impairments, such as acute lumbar strains, shoulder periarthritis, and soft tissue injuries. The mechanism underlying the effectiveness of motion-style acupuncture treatment for acute/subacute NSLBP primarily involves elevating pain thresholds, altering or inhibiting pain signal transmission, and the synergistic pain relief achieved through acupuncture and movement [31, 32]. Meta-analysis findings from Fei et al. [33••] indicated that motion-style acupuncture treatment effectively alleviates pain, lumbar functional impairments, and enhances lumbar range of motion (LROM) and overall efficacy rates (positive control or blank control/placebo). Our subgroup analysis of the 14 included studies highlighted that motion-style acupuncture treatments, particularly those involving three to six sessions, appeared to yield superior outcomes.

It is imperative to acknowledge that all included studies were inherently at high risk of performance and detection bias due to the absence of blinding. However, it is essential to recognize that both performance and detection bias are inherent to the types of interventions being compared. Furthermore, the evidence for most primary outcomes was determined to be of low or very low certainty. Nevertheless, when considering the minimum clinical significance value for primary outcomes, there is a discernible trend indicating the effectiveness of acupuncture, which holds clinical significance.

## Limitations

This study has several limitations. Due to the extensive utilization of acupuncture in Eastern countries, clinical research results in other languages (e.g., Japanese and Korean) could not be amalgamated, potentially impacting the conclusions based on language and region. The majority of the RCTs included in this meta-analysis were in Chinese; only three RCTs were in English, and two of them were conducted in China. Another limitation is the low methodological quality of the included studies, which diminished the strength of the resulting evidence. Furthermore, the absence of large-sample and multi-center randomized controlled trials imposes certain limitations on this meta-analysis.

## Conclusion

Based on the results of this systematic review, we are inclined to assert that acupuncture is more effective and safer than oral medication. In conclusion, this systematic review is poised to offer valuable guidance to clinicians treating acute/subacute NSLBP and potentially benefit the afflicted patients. We also hope that these findings will inspire further research in this domain.

### Supplementary Information

Below is the link to the electronic supplementary material.

Supplementary file1 (JPEG 40 KB)

Supplementary file2 (JPEG 33 KB)

Supplementary file3 (JPEG 13 KB)

Supplementary file4 (JPEG 13 KB)

Supplementary file5 (JPEG 43 KB)
