# Supplementary figures and images for: Acupuncture Versus Oral Medications for Acute/Subacute Non-Specific Low Back Pain: A Systematic Review and Meta-Analysis
Source: Curr Pain Headache Rep. 2024 Jan 8;28(6):489–500. doi: 10.1007/s11916-023-01201-7 (PMC11156714; doi:10.1007/s11916-023-01201-7)

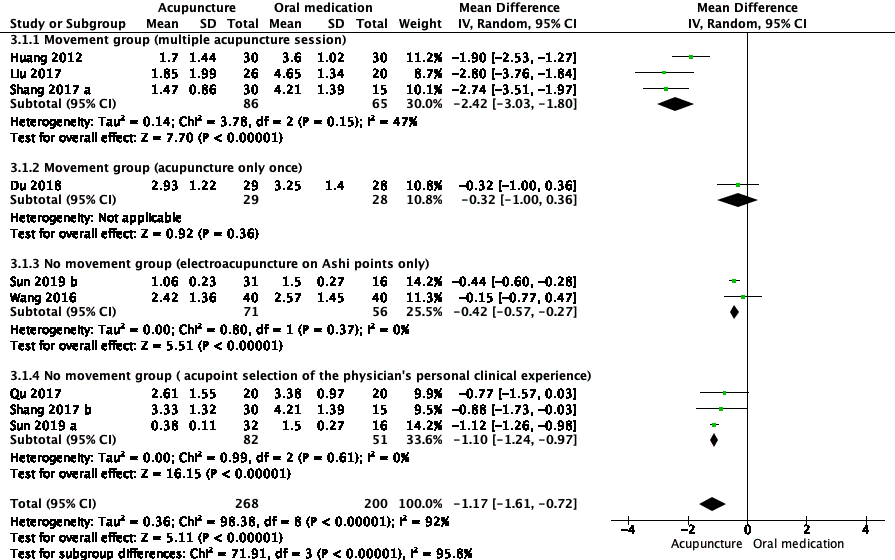

Supplement: Supplementary file 1 — Supplementary file1 (JPEG 40 KB) [file 11916_2023_1201_MOESM1_ESM.jpeg]

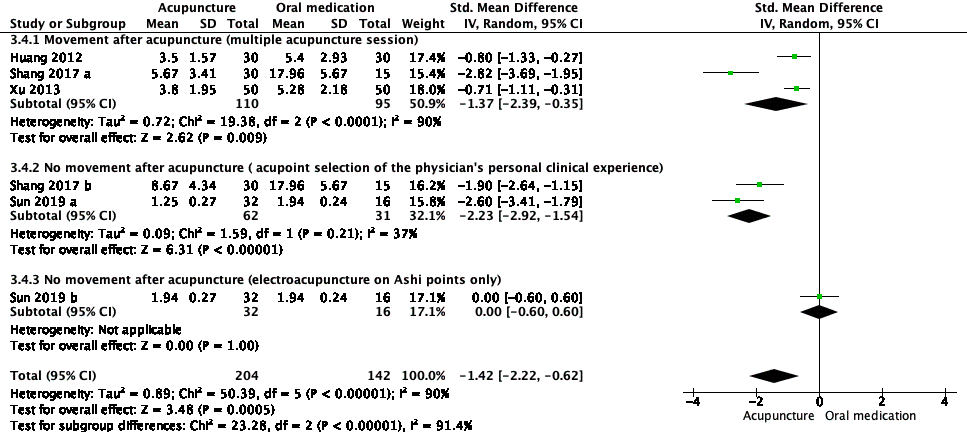

Supplement: Supplementary file 2 — Supplementary file2 (JPEG 33 KB) [file 11916_2023_1201_MOESM2_ESM.jpeg]

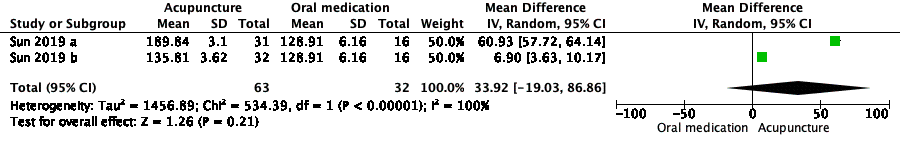

Supplement: Supplementary file 3 — Supplementary file3 (JPEG 13 KB) [file 11916_2023_1201_MOESM3_ESM.jpeg]

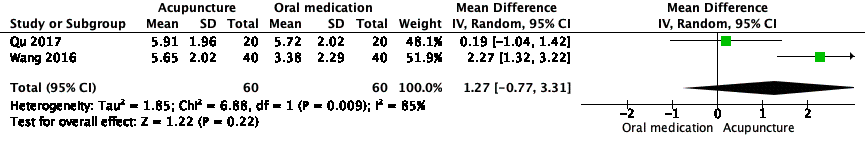

Supplement: Supplementary file 4 — Supplementary file4 (JPEG 13 KB) [file 11916_2023_1201_MOESM4_ESM.jpeg]

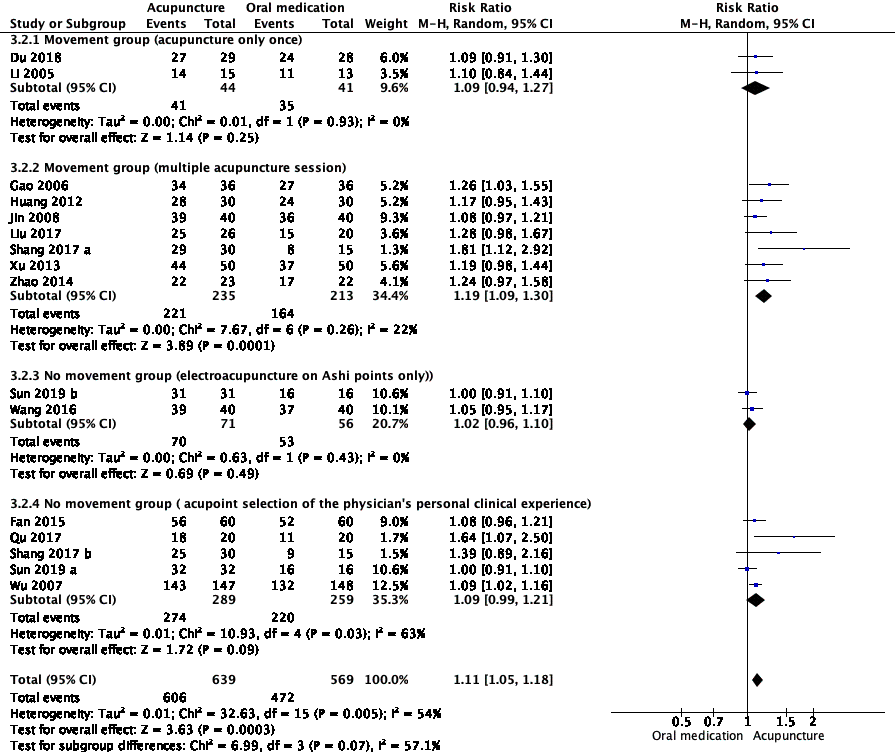

Supplement: Supplementary file 5 — Supplementary file5 (JPEG 43 KB) [file 11916_2023_1201_MOESM5_ESM.jpeg]
